# The Value of Neutrophil-to-Lymphocyte Ratio and Epicardial Adipose Tissue Thickness in Heart Failure With Preserved Ejection Fraction

**DOI:** 10.7759/cureus.42846

**Published:** 2023-08-02

**Authors:** Tugce Colluoglu, Yeşim Akın

**Affiliations:** 1 Cardiology, Karabük University, Karabük, TUR; 2 Cardiology, Karabuk University, Faculty of Medicine, Karabuk, TUR

**Keywords:** heart failure with preserved ejection fraction (hfpef), left ventricular function., hospitalisation for heart failure, neutrophil-to-lymphocyte ratio, epicardial adipose tissue thickness

## Abstract

Background

Using epicardial adipose tissue thickness (EATt) and neutrophil-to-lymphocyte ratio (NLR) as individual indicators provides beneficial insight into the prognosis of patients suffering from heart failure with preserved ejection fraction (HFpEF).

Aim

In our study, we aimed to evaluate whether the combined evaluation of NLR and EATt would provide an advantage for identifying high-risk HFpEF patients according to hospitalization for heart failure (HHF) and left ventricular diastolic dysfunction (LVDD).

Method

A total of 168 outpatients with HFpEF were retrospectively analyzed. The predictive performance of two inflammatory variables was assessed by the receiver operating characteristic curve and a one-way analysis of variance (ANOVA) test. The patients were stratified into three distinct risk categories based on the established cut-off values for EATt and NLR as follows: Group I, high risk; Group II, middle risk; and Group III, low risk.

Results

Patients in Group I had the highest risk for HHF and the presence of LVDD (p=0.001 for HHF, p=0.011 for LVDD). Patients in Group I also exhibited more symptomatic and a greater number of comorbidities. In Group I, more structural remodeling (enlarged left ventricular end-systolic volume index (LVESVI) and left atrial volume index (LAVI)) and associated signs of increased intracardiac pressure (elevated E/A ratio, medial E/e’) were observed.

Conclusion

The results of our study indicate that the use of both EATt and NLR among patients with HFpEF may provide better accuracy in predicting HHF and LVDD compared to the use of either EATt or NLR alone.

## Introduction

Heart failure with preserved ejection fraction (HFPEF) has become increasingly recognized as a significant public health concern on a worldwide basis [1]. The prevalence of HFpEF accounts for half of all heart failure cases and is projected to increase by approximately 50% by 2035 [2,3].

Systemic inflammation is believed to exert a significant influence on the pathogenesis of HFpEF. It has been observed that a prolonged inflammatory response can expedite the progression of HFpEF [1,4] and serve as a sign of the prognosis [5,6]. Simultaneously, systemic inflammatory mechanisms play an important role in increasing epicardial adipose tissue (EAT) and changing the nature of its secretion towards adipocytokines, which are pro-inflammatory [7-9]. These adipokines promote cardiomyocyte stiffness, coronary endothelial dysfunction, and myocardial fibrosis, all of which contribute to the development of HFpEF [10-12].

An increased EAT thickness (EATt), as detected by 2D transthoracic echocardiography (2D-TTE), is indicative of the accumulation of EAT [13]. Increased EATt has demonstrated an association with left ventricular diastolic dysfunction (LVDD) in pediatric patients, and augmentation of EATt in HFpEF is linked to unfavorable outcomes [13,14].

The neutrophil-to-lymphocyte ratio (NLR) is a convenient and accessible inflammatory marker that can be utilized for risk stratification in HFpEF [15-17]. As a potential marker for determining risk stratification in HFpEF, NLR potentially serves as a prognostic indicator for short- and long-term outcomes in patients diagnosed with HFpEF [17-19].

The use of EATt and NLR as individual markers may offer beneficial perspectives regarding the prognostic evaluation of patients diagnosed with HFpEF [20,21]. Nonetheless, there is an absence of scientific evidence on the concomitant use of these two markers, which may provide a more precise evaluation of the patient’s susceptibility to adverse outcomes. Therefore, the aim of this study was to assess the use of the combination of EATt and NLR as a predictive tool for one-year hospitalization for heart failure (HHF) and LVDD among patients diagnosed with HFpEF.

## Materials and methods

Study population

**Figure 1:**
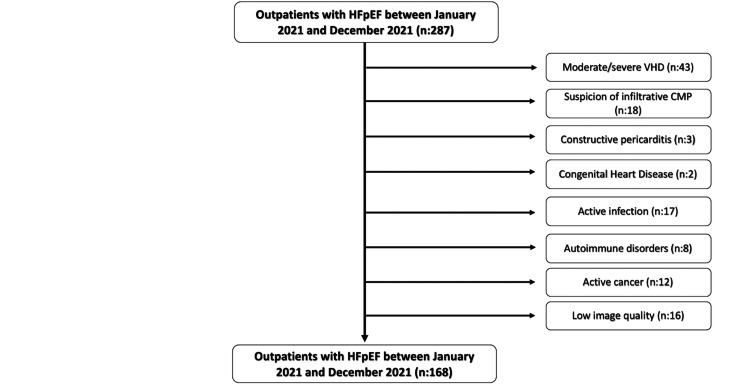
Flow chart for the selection of our study patients with heart failure with preserved ejection fraction HFpEF, heart failure with preserved ejection fraction; VHD, valvular heart diseases; CMP, cardiomyopathy

We retrospectively investigated 287 consecutive patients with preserved ejection fraction at the first ambulatory visit between January 2021 and December 2021 at Karabuk University, Faculty of Medicine, Department of Cardiology. The diagnosis of HFpEF was confirmed according to the 2021 European Society of Cardiology guidelines for the diagnosis and treatment of acute and chronic heart failure [22]. HFpEF was defined as the presence of symptoms (New York Heart Association (NYHA) ≥ II), increased brain natriuretic peptide (BNP) levels (BNP >35 pg/mL), and structural changes such as elevated left atrial volume index (LAVI) of >34 mL/m^2^, left ventricular hypertrophy, and/or functional changes, including E/e’>13 on transthoracic echocardiography (TTE). The major exclusion criteria were as follows: moderate to severe valvular heart disease as assessed by TTE (n=43), suspicion of infiltrative cardiomyopathy (n=18) and constructive pericarditis (n=3), congenital heart disease (n=2), active infection (n=17), documented history of autoimmune disorders (n=8), active cancer (n=12), as well as low image quality for echocardiographic analysis (n=16). As a result, 168 outpatients with HFpEF were included in this study (Figure 1). Electronic medical records were utilized to obtain the patients’ baseline clinical characteristics, standard laboratory parameters, and medication information. Routine biochemical tests, such as blood urine nitrogen (BUN), creatinine, estimated glomerular filtration rate (eGFR), Na+, K+, CI-, Mg++, Ca++, C-reactive protein (CRP), erythrocyte sedimentation rate (ESR), brain natriuretic peptide (BNP), triglycerides, and complete blood counts, including white blood cells (WBC), neutrophils, and lymphocytes, were obtained at the first ambulatory visit. The NLR was calculated by dividing the count of neutrophils by the count of lymphocytes, both of which were obtained from the same blood sample. The study was performed to conform with the Declaration of Helsinki and approved by the ethics committee of Karabük University, Faculty of Medicine.

Two-dimensional (2D) transthoracic echocardiographic image acquisition

**Figure 2:**
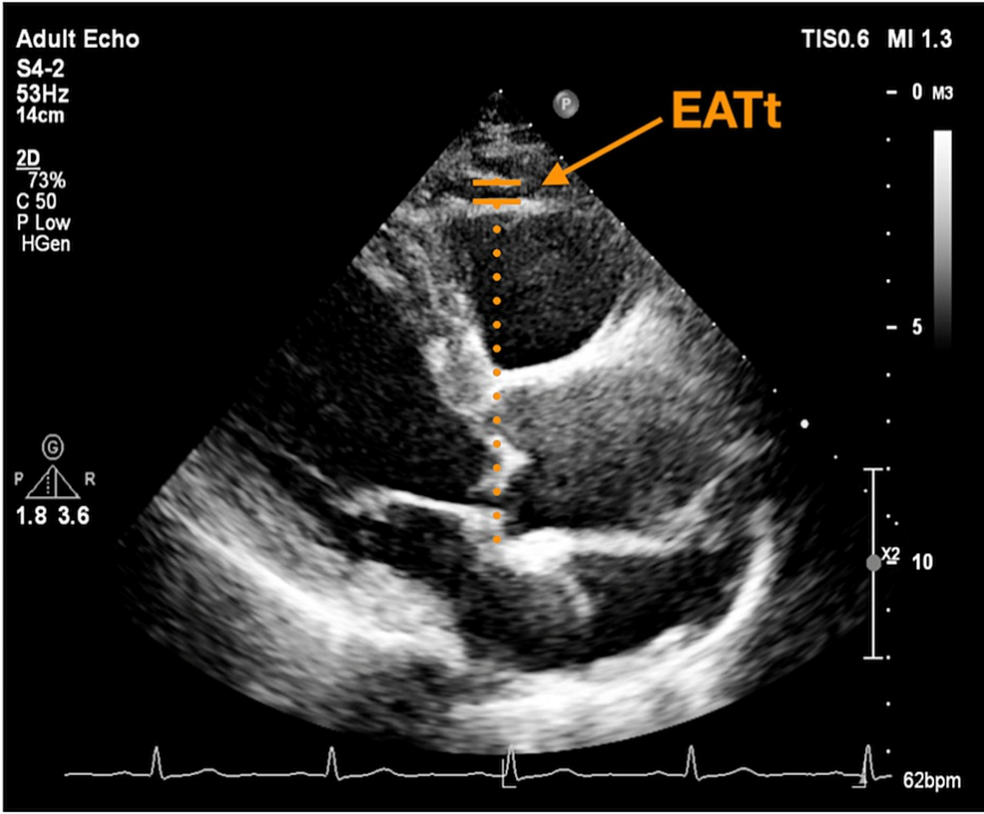
How to measure epicardial adipose tissue thickness (EATt) from the parasternal long-axis view

All echocardiographic examinations were performed with the Philips EPIQ-7C Ultrasound System for Cardiology (Andover, Massachusetts) with an X5-1 probe. All echocardiographic data were stored for offline analysis. 2D datasets were analyzed using the dedicated software. Interventricular septum dimension (IVSd), posterior wall dimension (PWd), left ventricular end-systolic volume (LVESV), and end-diastolic volume (LVEDV) were obtained through the analysis of 2D datasets. The measurement of EATt was done by using the reference line running through the right ventricular free wall and the aortic annulus. Then, the vertical length between the right ventricular free wall and the parietal pericardium was measured as EATt at end-diastole in three cardiac cycles (Figure 2) [23]. Left ventricular ejection fraction (LVEF) was measured using the modified biplane Simpson’s method. Left atrial volume (LAV) was measured using the biplane area-length method. LVEDVI, LVESVI, and LAVI were calculated by dividing LVEDV, LVESV, and LAV by body surface area, respectively. Early diastolic velocity (E wave) and late diastolic velocity (A wave) for the mitral valve (MV) and for the tricuspid valve were measured using pulsed wave (PW) Doppler from the apical four-chamber view, respectively. Peak longitudinal systolic velocity (Sm), peak longitudinal early diastolic velocity (e’), and peak longitudinal late diastolic velocity (a’) were measured using tissue Doppler imaging from the lateral and septal mitral annulus and the lateral tricuspid annulus, respectively. Left ventricular diastolic dysfunction in the left ventricle with preserved ejection fraction was defined according to the 2016 American Society of Echocardiography guidelines for the Evaluation of Left Ventricular Diastolic Function by Echocardiography [24]. Two experienced cardiologists independently interpreted all echocardiographic images. The inter- and intra-observer variabilities in echocardiographic measurements were 97% and 98%, respectively.

The ascertainment of one-year hospitalization for heart failure

We investigated the one-year follow-up of HHF after the first outpatient visit. The one-year HHF was determined from electronic medical records. The criteria for HHF were defined as follows: (1) the presence of pulmonary and/or systemic congestion findings; (2) the need for intravenous diuretics; (3) a BNP level of 100 pg/mL or higher; and/or (4) the presence of low cardiac output findings.

The definition of comorbidity burden

Comorbidities were defined based on physician documentation and a review of the medical records. Comorbidity burden was defined by a simple score based on the assignment of the following comorbidities (one point each): obesity, coronary artery disease, hypertension, hyperlipidemia, diabetes mellitus, atrial fibrillation, and chronic renal disease.

Statistical analysis

Continuous variables were assessed for normality using the Kolmogorov-Smirnov test. Continuous variables were presented as mean ± standard deviation or median (interquartile range (IQR)). Categorical variables were expressed as numbers and percentages. Continuous variables with a normal distribution were compared using the student’s t-test, and continuous variables with a non-normal distribution were compared using the Mann-Whitney U test. Categorical variables were compared using the chi-square test. Receiver operating characteristics curve (ROC) analysis was examined to determine optimal cut-off values of EATt and NLR for HHF. Patients were classified by the cut-off values of EATt and NLR. Analysis of variance (ANOVA) was used to compare the groups obtained from the cut-off values of EATt and NLR according to HHF and LVDD. A value of p<0.05 was considered statistically significant. The data analysis was performed with the SPSS 25.0 software program (IBM Corp., Armonk, NY).

## Results

**Figure 3:**
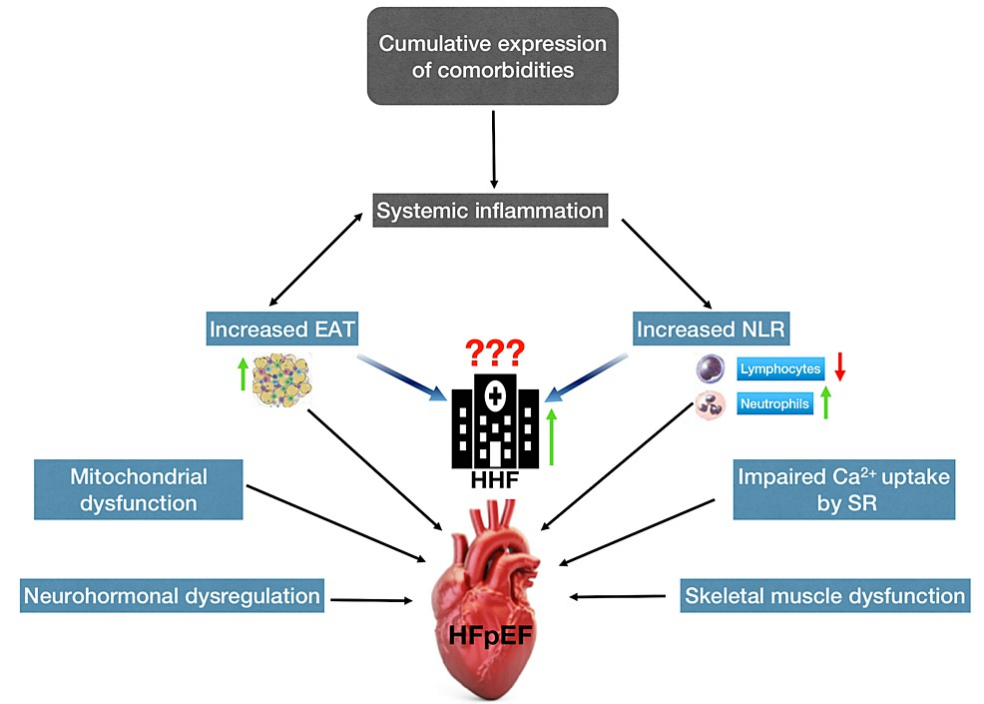
Graphical abstract Comorbidities-induced systemic inflammation leads to increased epicardial adipose tissue and neutrophil-to-lymphocyte ratio. Two proinflammatory variables play a major role in the development of heart failure with preserved ejection fraction. In addition, these markers play a contributory role in increased left ventricular filling pressure, which results in hospitalization for heart failure. Source: [7,15] EAT, epicardial adipose tissue; NLR, neutrophil-to-lymphocyte ratio; HHF, hospitalization for heart failure; SR, sarcoplasmic reticulum; HFpEF, heart failure with preserved ejection fraction.

Figure 3 presents a graphical abstract of the analysis. 

Baseline characteristics

The baseline characteristics of the total of 168 patients are shown in Table 1. In the overall study population, the mean age was 68.42±8.93 years, and 70% were female. Among the 168 study patients, 46 (27%) were hospitalized for heart failure. HHF patients had a worse functional status. Patients who developed HHF were characterized by a higher prevalence of chronic renal disease (73.9% vs. 30.3%, p=0.001), atrial fibrillation (30.4% vs. 14.8%, p=0.021), and a higher mean comorbidity burden (5.08±1.11 vs. 4.28±1.52, p=0.001). Heart rate was higher (82.13±18.75 bpm vs. 74.57±12.28 bpm, p=0.003), and beta-blockers, mineralocorticoid receptor antagonists (MRA), sodium-glucose co-transporter 2 inhibitors (SGLT2i), and furosemide were prescribed more often in patients who developed HHF (Table 1).

**Table 1:** Baseline characteristics of the study population HHF, hospitalization for heart failure; BMI, body mass index; NYHA, New York Heart Association; ACEI, angiotensin-converting enzyme inhibitors; ARB, angiotensin receptor blockers; MRA, mineralocorticoid receptor antagonists; SGLT2i, sodium-glucose cotransporter 2 inhibitors; CaCB, calcium channel blockers

Variables	Study population (n= 168)	HHF (+) (n= 46)	HHF (-) (n= 122)	p
Age (years)	68.42 ± 8.93	70.34 ± 8.85	67.70 ± 8.88	0.087
Sex (M/F)	50/118	16/30	34/88	0.382
BMI (kg/m^2^)	32.13 ± 6.01	33.09 ± 6.13	31.76 ± 5.95	0.202
Heart rate (bpm)	76.64 ± 14.68	82.13 ± 18.75	74.57 ± 12.28	0.003
Systolic blood pressure (mmHg)	138.27 ± 21.56	131.69 ± 20.30	140.80 ± 21.57	0.014
Diastolic blood pressure (mmHg)	81.77 ± 13.36	77.78 ± 12.66	83.27 ± 13.35	0.017
NYHA class II (%)	134 (79.8)	26 (56.5)	108 (88.5)	0.001
NYHA class III (%)	30 (17.9)	16 (34.8)	14 (11.5)	0.001
NYHA class IV (%)	4 (2.4)	4 (8.7)	0 (0.0)	0.001
Hypertension (%)	132 (78.6)	36 (76.1)	96 (78.7)	0.717
Hyperlipidemia (%)	76 (45.2)	22 (47.8)	54 (44.3)	0.679
Diabetes mellitus (%)	72 (42.9)	18 (39.1)	54 (44.3)	0.549
Chronic renal disease (%)	71 (42.3)	34 (73.9)	37 (30.3)	0.001
Coronary artery disease (%)	68 (40.5)	22 (47.8)	46 (37.7)	0.233
Atrial fibrillation (%)	32 (19.0)	14 (30.4)	18 (14.8)	0.021
Comorbidity burden	4.50 ± 1.46	5.08 ± 1.11	4.28 ± 1.52	0.001
Beta-blockers (%)	134 (79.8)	42 (91.3)	92 (75.4)	0.022
ACEI or ARB (%)	120 (71.4)	30 (65.2)	90 (73.8)	0.274
MRA (%)	50 (29.8)	20 (43.5)	30 (24.6)	0.017
SGLT2i (%)	36 (21.4)	16 (34.8)	20 (16.4)	0.010
Torasemide (%)	14 (8.3)	6 (13.0)	8 (6.6)	0.175
Furosemide (%)	60 (35.7)	30 (65.2)	30 (24.6)	0.001
CaCB (%)	64 (38.1)	14 (30.4)	50 (41.0)	0.209

The results of the complete blood count evaluations indicate that patients who experienced HHF exhibited a higher median NLR (3.61 (2.74-5.40) vs. 2.33 (1.71-2.95), p=0.001), WBC (8.76x109/L (7.00-10.80) vs. 7.20x109/L (6.00-8.78), p=0.001), neutrophil count (5.40x109/L (4.40-8.10) vs. 4.40x109/L (3.55-5.66), p=0.001), and lower mean lymphocyte count (1.74±0.84x109/L vs. 1.99±0.59x109/L, p=0.034) (Table 2). In comparison to patients who did not experience HHF, the median value of EATt was higher in patients who did experience HHF (11.0 mm (9.2-13.2) vs. 8.8 mm (7.2-9.9), p=0.001) (Table 2).

**Table 2:** Laboratory and echocardiographic parameters of the study population HHF, hospitalization for heart failure; BUN, blood urine nitrogen; Cr, creatinine; eGFR, estimated glomerular filtration rate; CRP, C-reactive protein; ESR, erythrocyte sedimentation rate; BNP, brain natriuretic peptide; WBC, white blood count; NLR, neutrophil to lymphocyte ratio; EAT, epicardial adipose tissue; LVEF, left ventricular ejection fraction; LVEDVI, left ventricular end-diastolic volume index; LVESVI, left ventricular end-systolic volume index; IVSd, interventricular septum dimension; PWd, posterior wall dimension; LAVI, left atrial volume index; RVd, right ventricular dimension; MV, mitral valve; TV, tricuspid valve; LV, left ventricle; RV, right ventricle

Variables	Study population (n= 168)	HHF (+) (n= 46)	HHF (-) (n= 122)	p
BUN (mg/dl)	41.00 (33.00-54.00)	48.00 (40.00-69.00)	39.00 (30.00-48.25)	0.001
Cr (mg/dl)	0.90 (0.70-1.10)	1.00 (0.90-1.40)	0.81 (0.68-1.10)	0.001
eGFR (ml/min/1.73 m^2^)	70.51 ± 24.21	57.23 ± 20.19	75.51 ± 23.77	0.001
Na^+^ (mEq/L)	139.40 ± 2.42	138.95 ± 2.53	139.57 ± 2.37	0.142
K^+^ (mEq/L)	4.53 ± 0.50	4.54 ± 0.65	4.53 ± 0.43	0.947
Cl^-^ (mEq/L)	101.73 ± 3.54	99.78 ± 3.89	102.46 ± 3.11	0.001
Mg^++^ (mg/dl)	1.91 ± 0.27	1.90 ± 0.37	1.92 ± 0.22	0.646
Ca^++ ^(mg/dl)	9.37 ± 1.13	9.08 ± 0.56	9.48 ± 0.44	0.036
Triglyceride (mg/dL)	137.00 (107.25-206.75)	131.00 (103.00-206.00)	140.00 (107.75-209.25)	0.776
ESR (mm/h)	23.00 (14.00-35.75)	28.00 (14.00-37.00)	22.50 (13.00-32.75)	0.068
CRP (mg/L)	3.82 (7.60-12.03)	9.90 (5.90-18.00)	5.50 (3.47-12.00)	0.007
BNP (pg/ml)	135.30 (69.03-196.72)	136.05 (72.73-244.86)	126.84 (68.60-183.00)	0.280
Haemoglobin (g/dl)	12.63 ± 1.38	12.66 ± 1.40	12.62 ± 1.38	0.881
WBC (10^9^/L)	7.50 (6.20-9.07)	8.76 (7.00-10.80)	7.20 (6.00-8.78)	0.001
Neutrophile count (10^9^/L)	4.55 (3.80-6.20)	5.40 (4.40-8.10)	4.40 (3.55-5.66)	0.001
Lymphocyte count (10^9^/L)	1.92 ± 0.68	1.74 ± 0.84	1.99 ± 0.59	0.034
NLR	2.59 (1.89-3.63)	3.61 (2.74-5.40)	2.33 (1.71-2.95)	0.001
NLR ≥2.83 (%)	73 (43.5)	36 (78.3)	37 (30.3)	0.001
Platelet count (10^9^/L)	254.00 (213.25-298.00)	251.00 (206.00-306.00)	254.00 (213.75-296.50)	0.887
EAT thickness (mm)	9.2 (7.8-11.0)	11.0 (9.2-13.2)	8.8 (7.2-9.9)	0.001
EAT thickness ≥9.45 mm (%)	79 (47)	33 (71.7)	46 (37.7)	0.001
LVEF (%)	55.20 (51.60-58.40)	55.50 (51.00-57.30)	55.00 (51.70-59.00)	0.229
LVEDVI (ml/m^2^)	42.21 (35.38-49.17)	42.16 (29.47-47.38)	42.21 (35.71-49.39)	0.243
LVESVI (ml/m^2^)	18.97 (14.19-22.33)	18.97 (12.57-22.54)	18.92 (14.60-22.31)	0.378
IVSd (cm)	1.21 ± 0.23	1.26 ± 0.28	1.19 ± 0.20	0.127
PWd (cm)	0.92 ± 0.15	0.92 ± 0.17	0.92 ± 0.14	0.988
LAVI (ml/m^2^)	36.45 (24.13-42.42)	38.42 (25.49-45.91)	35.57 (23.94-40.75)	0.309
RVd (cm)	2.60 (2.38-2.87)	2.51 (2.39-2.80)	2.61 (2.34-2.90)	0.856
MV E wave (cm/s)	85.00 (70.80-101.00)	85.00 (61.60-114.00)	85.00 (71.80-99.40)	0.966
MV A wave (cm/s)	87.80 (69.70-102.25)	77.40 (58.10-103.25)	88.80 (70.30-102.25)	0.206
MV E/A ratio	0.88 (0.74-1.04)	0.76 (0.69-1.29)	0.89 (0.76-1.03)	0.489
TV E wave (cm/s)	51.65 (44.00-59.20)	51.40 (45.10-63.00)	51.90 (43.20-58.70)	0.356
TV A wave (cm/s)	51.40 (44.07-59.20)	57.40 (48.75-59.27)	50.20 (43.07-58.82)	0.096
TV E/A ratio	1.00 (0.80-1.10)	0.90 (0.71-1.15)	1.00 (0.80-1.10)	0.246
LV- lateral Sm (cm/s)	6.96 (6.09-8.27)	6.09 (5.66-7.40)	7.18 (6.29-8.46)	0.001
LV-medial Sm (cm/s)	6.31 (5.44-6.96)	6.42 (4.80-6.85)	6.31 (5.44-7.07)	0.372
LV- lateral E/e’	9.71 (7.56-13.08)	11.33 (8.17-13.09)	9.40 (7.52-12.24)	0.149
LV- medial E/e’	12.89 (11.23-18.24)	14.56 (12.44-18.80)	12.60 (10.81-16.96)	0.028
RV- lateral Sm (cm/s)	12.40 (11.10-13.90)	12.40 (10.10-15.00)	12.40 (11.30-13.85)	0.681

Predictive value of epicardial adipose tissue thickness and neutrophil-to-lymphocyte ratio for heart failure hospitalization

**Figure 4:**
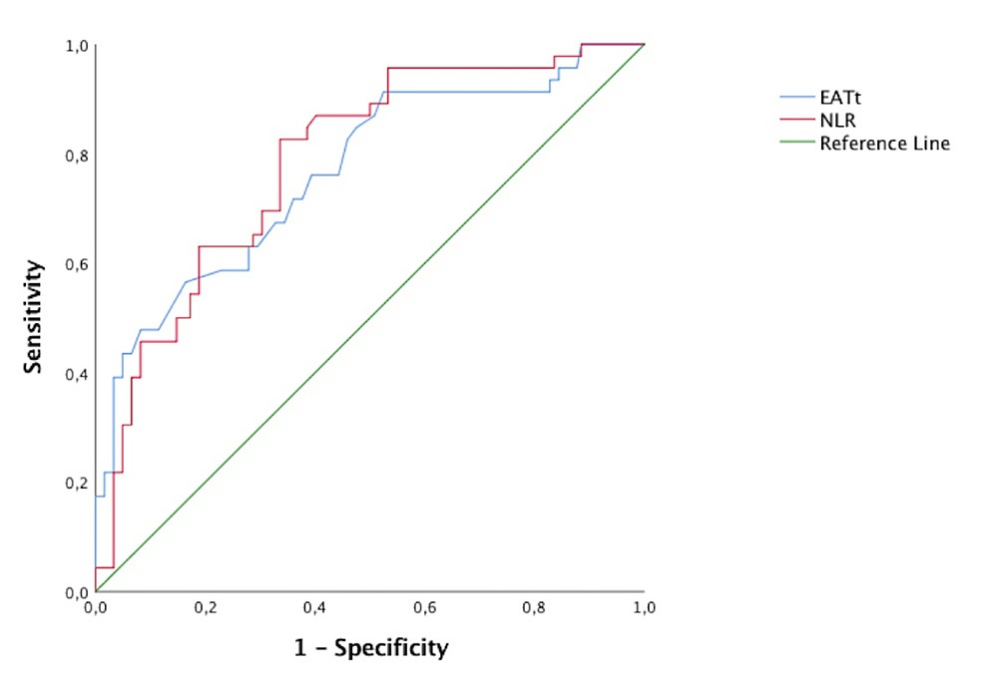
Receiver operating characteristic curve of the epicardial adipose tissue thickness and neutrophil-to-lymphocyte ratio in predicting hospitalization for heart failure EATt, epicardial adipose tissue thickness; NLR, neutrophil-to-lymphocyte ratio

Receiver operating characteristic curves were constructed to assess the ability of the EATt and NLR to predict HHF. The area under the ROC curve (AUC) of EATt and NLR in HFpEF patients was 0.767 and 0.785 (p=0.001 for EATt and NLR), respectively (Figure 4, Table 3). EATt ≥9.45 mm predicted HHF with a specificity of 64% and sensitivity of 71%. NLR ≥2.83 predicted HHF with a specificity of 70% and sensitivity of 70%.

**Table 3:** Receiver operating characteristic curve of the epicardial adipose tissue thickness and neutrophil-to-lymphocyte ratio in predicting hospitalization for heart failure EATt, epicardial adipose tissue thickness; NLR, neutrophil-to-lymphocyte ratio

Test results variable(s)	Area	Std. Error	Asymptotic Sig.	Lower bound	Upper bound
EATt	0.767	0.043	<0.001	0.683	0.850
NLR	0.785	0.038	<0.001	0.711	0.860

Findings based on the cut-off values of epicardial adipose tissue thickness and neutrophil-to-lymphocyte ratio

Patients were categorized into three risk groups based on the cut-off values of EATt and NLR, which predict HHF (high risk: EATt ≥9.45 mm and NLR ≥2.83 (Group I), middle risk: EATt ≥9.45 mm and NLR <2.83 or EATt <9.45 mm and NLR ≥2.83 (Group II), low risk: EATt <9.45 mm and NLR <2.83 (Group III)).

**Figure 5:**
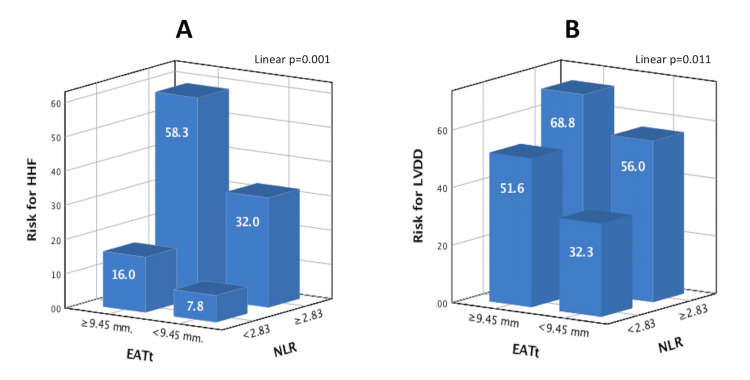
The risk of hospitalization for heart failure (A) and the presence of left ventricular diastolic dysfunction (B) based on the cut-off values of epicardial adipose tissue thickness and neutrophil-to-lymphocyte ratio HHF, hospitalization for heart failure; LVDD, left ventricular diastolic dysfunction; EATt, epicardial adipose tissue thickness; NLR, neutrophil-to-lymphocyte ratio

Our results showed that NLR ≥2.83 and EATt ≥9.45 mm were associated with significantly higher rates of HHF, separately. We further investigated the predictive value of the combination of NLR and EATt. One-way analysis of the variance test also found that the combination of NLR ≥2.83 and EATt ≥9.45 mm had the highest risk for HHF and LVDD (p=0.001 for HHF, p=0.011 for LVDD) (Figures 5A, 5B). Patients in Group I were more symptomatic, with more frequent chronic renal disease and coronary artery disease, a higher comorbidity burden, and a higher rate of diuretic treatment assignment (Table 4).

**Table 4:** Comparison of clinical characteristics based on the cut-off values of NLR and EAT thickness BMI, body mass index; NYHA, New York Heart Association; ACEI, angiotensin-converting enzyme inhibitors; ARB, angiotensin receptor blockers; MRA, mineralocorticoid receptor antagonists; SGLT2i, sodium-glucose cotransporter 2 inhibitors; CaCB, calcium channel blockers *Group I: NLR ≥2.83 and EAT ≥9.45; Group II: NLR ≥2.83 and EAT <9.45 or NLR <2.83 and EAT ≥9.45; Group III: NLR <2.83 and EAT <9.45

Variables	Group I (n= 48)	Group II (n= 56)	Group III (n= 64)	p
Age (years)	69.5 ± 10.76	68.14 ± 9.23	67.87 ± 7.02	0.611
Sex (M/F)	17/31	20/36	13/51	0.110
BMI (kg/m^2^)	32.03 ± 5.28	32.33 ± 6.04	32.02 ± 6.52	0.956
Heart rate (bpm)	74.35 ± 12.41	80.19 ± 18.32	75.25 ± 18.08	0.081
Systolic blood pressure (mmHg)	139.84 ± 22.50	138.37 ± 25.79	137.06 ± 16.47	0.801
Diastolic blood pressure (mmHg)	78.18 ± 15.74	82.41 ± 13.80	83.90 ± 10.34	0.073
NYHA class II (%)	26 (54.2)	52 (92.9)	56 (87.5)	0.001
NYHA class III (%)	18 (37.5)	4 (7.1)	8 (12.5)	0.001
NYHA class IV (%)	4 (8.3)	0 (0.0)	0 (0.0)	0.001
Hypertension (%)	38 (79.2)	44 (78.6)	50 (78.1)	0.991
Hyperlipidemia (%)	26 (54.2)	21 (37.5)	29 (45.3)	0.235
Diabetes mellitus (%)	22 (45.8)	26 (46.4)	24 (37.5)	0.545
Chronic renal disease (%)	34 (70.8)	23 (41.1)	14 (21.9)	0.001
Coronary artery disease (%)	29 (60.4)	19 (33.9)	20 (31.3)	0.004
Atrial fibrillation (%)	15 (31.3)	10 (17.9)	7 (10.9)	0.025
Hospitalization for heart failure (%)	28 (58.3)	13 (23.2)	5 (7.8)	0.001
Comorbidity burden	5.10 ± 1.25	4.41 ± 1.48	4.14 ± 1.47	0.001
Beta-blockers (%)	40 (83.3)	46 (82.1)	48 (75.0)	0.478
ACEI or ARB (%)	29 (60.4)	43 (76.8)	48 (75.0)	0.133
MRA (%)	20 (41.7)	16 (28.6)	14 (21.9)	0.074
SGLT2i (%)	14 (29.2)	13 (23.2)	9 (14.1)	0.144
Torasemide (%)	8 (16.7)	2 (3.6)	4 (6.3)	0.041
Furosemide (%)	23 (47.9)	21 (37.5)	16 (25.0)	0.041
CaCB (%)	19 (39.6)	24 (42.9)	21 (32.8)	0.511

Group I had a worse renal function test, and a larger median BNP level, CRP, NLR, and neutrophil count. It also had a lower mean lymphocyte count than the other groups (Table 5). Patients in Group I exhibited significantly worse systolic and diastolic function as compared with Group II and Group III (Table 5).

**Table 5:** Comparison of laboratory and echocardiographic parameters based on the cut-off values of NLR and EAT thickness BUN, blood urine nitrogen; Cr, creatinine; eGFR, estimated glomerular filtration rate; CRP, C-reactive protein; BNP, brain natriuretic peptide; WBC, white blood count; NLR, neutrophil to lymphocyte ratio; EAT, epicardial adipose tissue; LVEF, left ventricular ejection fraction; LVEDVI, left ventricular end-diastolic volume index; LVESVI, left ventricular end-systolic volume index; IVSd, interventricular septum dimension; PWd, posterior wall dimension; LAVI, left atrial volume index; RVd, right ventricular dimension; MV, mitral valve; TV, tricuspid valve; LV, left ventricle; RV, right ventricle * Group I: NLR ≥2.83 and EAT ≥9.45; Group II: NLR ≥2.83 and EAT <9.45 or NLR <2.83 and EAT ≥9.45; Group III: NLR <2.83 and EAT <9.45.

Variables	Group I (n= 48)	Group II (n= 56)	Group III (n= 64)	p
BUN (mg/dl)	48.00 (38.00-72.50)	42.50 (30.75-55.50)	37.00 (32.00-41.75)	0.001
Cr (mg/dl)	1.10 (0.90-1.40)	0.90 (0.70-1.10)	0.80 (0.64-1.00)	0.001
eGFR (ml/min/1.73 m^2^)	59.83 ± 27.16	73.47 ± 25.48	75.92 ± 17.67	0.001
Na^+^ (mEq/L)	139.22 ± 22.81	139.37 ± 2.53	139.56 ± 2.00	0.769
K^+^ (mEq/L)	4.64 ± 0.60	4.46 ± 0.56	4.52 ± 0.32	0.186
Cl^-^ (mEq/L)	100.62 ± 4.49	101.76 ± 3.15	102.53 ± 2.81	0.018
Mg^++^ (mg/dl)	1.91 ± 0.27	1.89 ± 0.33	1.93 ± 0.22	0.738
Ca^++ ^(mg/dl)	9.10 ± 1.47	9.62 ± 0.52	9.38 ± 1.19	0.084
Sedimentation (mm/h)	32.00 (21.50-43.75)	22.00 (13.25-35.75)	18.00 (12.00-31.00)	0.090
CRP (mg/L)	10.65 (5.30-15.75)	7.25 (4.05-12.00)	5.25 (3.30-10.72)	0.001
BNP (pg/ml)	197.26 (112.15-321.62)	145.81 (60.61-176.18)	112.60 (61.89-157.17)	0.001
Haemoglobin (g/dl)	12.27 ± 1.17	12.83 ± 1.51	12.72 ± 1.39	0.093
WBC (10^9^/L)	8.90 (7.02-11.17)	8.05 (6.02-9.35)	7.10 (6.00-8.20)	0.001
Neutrophile count (10^9^/L)	5.95 (4.40-8.17)	4.85 (3.92-6.12)	4.10 (3.12-4.50)	0.001
Lymphocyte count (10^9^/L)	1.48 ± 0.72	1.92 ± 0.61	2.25 ± 0.48	0.001
NLR	4.25 (3.07-6.41)	2.55 (2.04-3.58)	1.82 (1.38-2.29)	0.001
Platelet count (10^9^/L)	258.00 (244.75-321.75)	221.50 (191.25-298.00)	250.00 (213.75-292.00)	0.035
EAT thickness (mm)	12.35 (10.00-14.22)	9.60 (8.80-10.70)	7.80 (5.00-8.50)	0.001
LVEF (%)	53.50 (51.52-56.97)	56.80 (54.40-60.00)	55.75 (51.20-60.20)	0.025
LVEDVI (ml/m^2^)	42.67 (36.96-51.73)	41.89 (32.18-46.37)	40.75 (34.16-49.80)	0.159
LVESVI (ml/m^2^)	20.51 (16.62-24.11)	17.54 (13.97-21.28)	17.94 (14.02-21.37)	0.024
IVSd (cm)	1.21 ± 0.28	1.26 ± 0.21	1.17 ± 0.18	0.098
PWd (cm)	0.92 ± 0.18	0.93 ± 0.13	0.90 ± 0.13	0.451
LAVI (ml/m^2^)	38.77 (30.58-42.60)	34.47 (24.13-42.73)	34.90 (22.73-40.75)	0.024
RVd (cm)	2.67 (2.48-2.84)	2.64 (2.40-2.90)	2.47 (2.33-2.81)	0.215
MV E wave (cm/s)	85.40 (71.07-114.00)	84.90 (64.75-104.75)	83.40 (71.80-94.40)	0.584
MV A wave (cm/s)	74.70 (66.50-103.25)	87.55 (66.90-108.50)	92.20 (71.80-102.00)	0.301
MV E/A ratio	1.43 (0.70-1.96)	1.08 (0.73-1.44)	0.85 (0.76-0.96)	0.024
TV E wave (cm/s)	55.00 (44.15-63.10)	51.90 (43.92-58.70)	50.20 (43.20-54.20)	0.060
TV A wave (cm/s)	54.80 (46.10-59.27)	49.50 (41.77-60.60)	51.20 (43.67-56.20)	0.414
TV E/A ratio	0.98 (0.71-1.20)	1.00 (0.78-1.10)	1.00 (0.80-1.10)	0.932
LV- lateral Sm (cm/s)	6.74 (5.80-8.46)	6.78 (6.09-7.72)	7.40 (6.19-8.70)	0.122
LV-medial Sm (cm/s)	6.53 (5.11-7.18)	6.45 (5.27-7.51)	6.09 (5.61-6.64)	0.308
LV- lateral E/e’	9.37 (7.01-12.67)	10.98 (7.62-13.47)	8.74 (7.58-11.75)	0.350
LV- medial E/e’	13.28 (10.84-18.49)	12.29 (10.39-14.34)	6.74 (5.80-8.46)	0.016
RV- lateral Sm (cm/s)	12.40 (9.00-14.60)	12.80 (11.35-14.10)	12.15 (11.30-13.50)	0.482

## Discussion

The main findings from the current study were as follows: (1) patients who manifested HHF demonstrated higher EATt and NLR; (2) the optimal threshold values for EATt and NLR in predicting HHF were 9.45 mm and 2.83, respectively; and (3) the co-occurrence of EATt and NLR exceeding the established thresholds demonstrated the highest level of susceptibility to the development of HHF and LVDD.

The rise in EATt and NLR may have the potential to raise the probability of HHF due to the resultant elevation in intracardiac pressure and natriuretic peptides. A noteworthy observation pertaining to this finding is that a marked augmentation in both EAT mass and the inflammatory reaction was noted in the mouse model deficient in ACE2. As a result, there was a notable elevation in the left ventricular filling pressure [25,26]. This observation may indicate that increased EATt and elevated NLR correlate with more severe symptoms, worse exercise capacity, and poorer cardiopulmonary performance among HFpEF patients [27]. Our study also consistently revealed that patients with high EATt (≥9.45 mm) and high NLR (≥2.45) had more impaired functional capacity with higher LV medial E/e’, a noninvasive indicator of LV filling pressure. These data highlight that strategies to decrease the signs of an inflammatory response, such as EATt and NLR, may also reduce the progression of hemodynamic impairment to improve HHF in patients with HFpEF.

Our findings demonstrated for the first time the deleterious effects of inflammation on LV diastolic function with the combined use of two inflammatory variables. The current study revealed that patients with elevated levels of both EATt and NLR exhibited notably poor LV diastolic function. The present finding implies that the concurrent elevation of EATt and NLR could potentially act as a trigger for LVDD or indicate an intensified inflammatory reaction linked to LVDD. Elevated EATt has been observed to promote the secretion of adipokines with pro-inflammatory properties, thereby potentially contributing to an increase in NLR. This may lead to myocardial fibrosis, LVDD, and impaired relaxation [28,29].

Increased EATt and elevated NLR as a sign of aggravated inflammation may serve as a crucial mechanism involved in facilitating LV structural remodeling [30,31], which in turn causes left atrial (LA) electrical remodeling over time in HFpEF patients [32-34]. Given the mechanistic relationship between atria and ventricles, an increase in LV filling pressures may be paralleled by LA dilatation and may contribute to generating a proarrhythmogenic substrate in patients with a high-grade pro-inflammatory state. Therefore, the results of our study highlight that increased EATt and elevated NLR may enhance the MV E/A ratio and LV medial E/e’, thereby accelerating the development of structural remodeling, such as LV remodeling, LA dilatation, and promoting the emergence of electrophysiological substrates for atrial fibrillation (AF) in HFpEF (Table 4 and Table 5).

Comorbidity burden can be a significant marker of inflammation levels in the HFpEF [16,35]. Patients with multiple comorbidities are more likely to have elevated levels of NLR and increased EAT volume [17,36,37]. Therefore, the observed association between systemic inflammation and increased NLR and EATt may be consistent with the notion that patients with HFpEF who exhibit higher NLR and EATt suffer from a greater number of comorbidities. The presence of a greater comorbidity burden, indicative of an elevated systemic inflammation response, may potentially play a role in the pathophysiology of HFpEF via similar mechanisms such as inflammation. This inflammatory pathway may contribute to the development of cardiac fibrosis and impaired diastolic function [29]. Overall, comorbidity burden may seem to indicate the association between elevated inflammatory response and hemodynamic derangements, as well as their resultant effects in patients with HFpEF.

Limitations

Our study is a single-center and retrospective study. Due to the relatively low number of patients, statistical findings are only arguably verified. We only assessed EATt in two dimensions by echocardiography. Although the determination of EAT volume by cardiac magnetic resonance is more accurate, there are also studies in which EATt was determined by echocardiography [38-40]. Therefore, we did not quantify the volume of EAT due to planimetric measurement. This study only included outpatients with HFpEF; it would have also been valuable to include inpatients with newly diagnosed HFpEF. In addition, we evaluated one-year outcomes after index admission. Further studies with larger sample sizes and longer follow-ups are needed to better verify the association between EATt and both inflammatory biomarkers and hard endpoints in HFpEF patients.

## Conclusions

The concomitant use of EATt and NLR may potentially yield a more accurate assessment of the susceptibility to HHF and the emergence of cardiac impairment in comparison with the individual use of EATt and NLR, which reflect systemic inflammation. This emphasizes the importance of addressing the underlying inflammation in the pathogenesis of HFpEF.
